# National Dental Association of the United States of America

**Published:** 1882-07

**Authors:** R. Finley Hunt

**Affiliations:** Washington, D. C.


### NATIONAL DENTAL ASSOCIATION OF THE UNITED STATES OF AMERICA.

The next meeting of the National Dental Association of the United States of America, will be held in Washington, D. C., in the lecture hall of the National Museum of the Smithsonian Institution, on the 3d, 4th, and 5th of August, 1882.

The meetings of the Association in Washington are quadrennial and international. A cordial invitation is extended to all members of the profession in this and other countries, to attend this meeting. R. Finley Hunt, D.D.S.
Washington, D. C. Secretary.

